# Reliability of the American Community Survey for unintentional drowning and submersion injury surveillance: a comprehensive assessment of 10 socioeconomic indicators derived from the 2006–2013 annual and multi-year data cycles

**DOI:** 10.1186/s40621-015-0065-0

**Published:** 2015-12-29

**Authors:** Nathaniel Bell, Bo Cai

**Affiliations:** 1 College of Nursing, University of South Carolina, 1601 Green St., Columbia, SC 29208 USA; 2 Department of Epidemiology and Biostatistics, Arnold School of Public Health, University of South Carolina, 915 Green St., Columbia, SC 29208 USA; 3 Department of Surgery, University of South Carolina, 2 Medical Dr., Columbia, SC 29203 USA

**Keywords:** American Community Survey, Injuries, Drowning, Social class, Risk

## Abstract

**Background:**

Our objective was to evaluate the reliability and predictability of ten socioeconomic indicators obtained from the 2006–2013 annual and multi-year ACS data cycles for unintentional drowning and submersion injury surveillance.

**Methods:**

Each indicator was evaluated using its margin of error and coefficient of variation. For the multi-year data cycles we calculated the frequency that estimates for the same geographic areas from consecutive surveys were statistically significantly different. Relative risk estimates of drowning-related deaths were constructed using the National Center for Health Statistics compressed mortality file. All analyses were derived using census counties.

**Results:**

Five of the ten socioeconomic indicators derived from the annual and multi-year data cycles produced high reliability CV estimates for at least 85 % of all US counties. On average, differences in socioeconomic characteristics for the same geographic areas for consecutive 3- and 5-year data cycles were unlikely to be caused by sampling error in only 17 % (5–89 %) and 21 % (5–93 %) of all counties. No indicator produced statistically significant relative risk estimates across all data cycles and survey years.

**Conclusions:**

The reliability of the annual and multi-year county-level ACS data cycles varies by census indicator. More than 75 % of the differences in estimates between consecutive multi-year surveys are likely to have occurred as a result of sampling error, suggesting that researchers should be judicious when interpreting overlapping survey data as reflective of real changes in socioeconomic conditions. Although no indicator predicted disparities in drowning-related injury mortality across all data cycles and years, further studies are needed to determine if these associations remain consistent at different geographic scales and for injury morbidity.

## Background

Drowning-related deaths continue to be a major public health problem in the United States (US). Between 2009 and 2013 it was the third most common type of injury-related death among children under five and the second most common among adolescents ages five to fourteen, accounting for 3488 child and adolescent deaths (Centers for Disease Control. Causes of Injury Death: Centers for Disease Control and Prevention & Control 2013). Over this same period it remained a top ten cause of all injury-related deaths among all persons under the age of 55.

Factors such as lower socioeconomic status, age, gender, ethnicity, alcohol, and living in a rural environment all increase risk of hospitalization or death from drowning (Brenner et al. 2001; Browne et al. 2003; Burrows et al. 2013; Giashuddin et al. 2009; Gilchrist & Parker 2014; Kim et al. 2007; Peek-Asa et al. 2004; Saluja et al. 2006; Thompson & Rivara 2000; Wright et al. 2013). Among rural populations, risks are attributable to greater access to open water sources, irrigation ditches, and watering troughs for livestock. Lack of adequate fencing and pool enclosures often characterize urban-related incidence rates, whereas factors such as low income are pervasive. These factors are routinely monitored and are part of numerous prevention and intervention initiatives (Cortes et al. 2006; Depczynski et al. 2009; Girasek 2011). Recent systematic reviews show that these factors remain indicative of variation in risk both in the US and abroad (Leavy et al. 2015a; Leavy et al. 2015b; Wallis et al. 2015).

However, US investigators tasked with monitoring drowning-related injuries now face a particular difficulty measuring its burden. This is because the 2010 replacement of the long form decennial census with the American Community Survey (ACS) fundamentally changed the structure of the geographic and socioeconomic data for the population. There are two primary differences between the ACS and the decennial census. First, the ACS is a rolling survey. Each month, approximately 250,000 households are interviewed using multiple survey mediums (US Census Bureau 2008) The survey responses are then aggregated into 1-year periods for annual release, with larger pools obtained prior to the release of the multi-year cycles. The structure and sample size of the ACS is in contrast to the decennial census, which obtained point-in-time responses from 1 out of 6 households every 10 years. Second, the ACS provides improved access to current socioeconomic information on the US population through the release of an annual, 3-year, and 5-year data cycles. The multi-year cycles are considered to be more reliable as they contain estimates from three and five times as many responses and represent a larger number of areas (US Census Bureau 2009) This is particularly relevant for subgroup questions that impact a smaller proportion of the population (e.g. female head of household with no husband and with children at home under 18 years of age) compared to the population profile questions (e.g. % male).

Accordingly, there are now two primary challenges attributed to the shift from the decennial census to the ACS. The first challenge pertains to the reliability of the ACS. For example, one study found that the margins of error (MOE) for certain Census Tracts increased over 80 % from the 2000 decennial census (Alexander 2002). Another study found that the MOE for the 5-year cycle was large enough to cause census tracts to move from highest into the lowest income quartile (Spielman et al. 2014). A more profound problem was that the variability predominated within marginalized areas. The second challenge pertains to coverage. For example, the annual data cycle is only released for geographic areas of at least 65,000 persons whereas the 3-year cycle is only available for areas of at least 20,000 persons. This effectively eliminates approximately 75 % of what are predominantly rural and non-metropolitan counties from annual surveillance, while providing current information for approximately 58 % of the population every 3 years.

One proposed strategy to circumvent limited access to current socioeconomic information from the ACS is to use consecutive multi-year surveys to represent ‘annual’ socioeconomic characteristics (US Census Bureau 2009). However, the fundamental challenge of this approach is that any difference in the estimates could be driven by data from non-overlapping survey years. For example, 80 % of the ACS estimates from the 2012 5-year data cycle (e.g. 2008–2012 surveys) overlap the 2013 release file (e.g. years 2009–2013). If used consecutively, 67 % of the estimates for consecutive 3-year data cycles overlap. As such, any comparison using overlapping survey periods must determine whether the observed differences are unlikely to have occurred by chance due to sampling error.

We examined the reliability and predictability of the annual and multi-year ACS data cycles for measuring socioeconomic disparities in drowning-related injury mortality across the US between 2006 and 2013 using county-level data. Our analysis included ten indicators previously used for quantifying socioeconomic disparities in drowning-related injury rates both in the US and abroad. Our primary objective was to record the amount of variability in the indicators when constructed using the annual and multi-year data cycles. Our secondary objective was to determine whether there was a particular data cycle and indicator that could facilitate better detection of differences in injury risk over time.

## Methods

This study was a retrospective cohort study of drowning-related injury mortality incidence calculated from the annual and multi-year ACS data cycles. Data regarding the injured population were obtained from the National Center for Health Statistics (NCHS) annual detailed mortality file for all US residents for the period 2006–2013. Drowning-related fatalities were specified using International Classification of Disease, Tenth Revision (ICD-10) cause of death codes, inclusive of W65-W74. Mortality and socioeconomic data files were linked using county identification numbers and evaluated as age adjusted rates. County geographic boundaries were selected for analysis because they are the finest administrative data available for the annual ACS data cycle. County boundaries are also robust for national injury and disease surveillance (Singh 2003; Wilkinson & Pickett 2008).

Reliability of the ACS data cycles was evaluated using the coefficient of variation (CV) for each estimate. The National Research Council defines a “reasonable standard of precision” as a CV percentage between 0 % and 12 % (National Research Council 2007). Here, we use a categorical rankings of “high reliability” (0–12 %), “moderate reliability” (12–40 %), and “low reliability” (>40 %) to assess the estimates (Environmental Systems Research Institute. The American Community Survey: An ESRI white paper: ESRI 2014). The standard error required to construct each CV was calculated using an adjustment factor of 95 % confidence. This requires multiplying the margin of error (MOE) for each estimate or its numerator/denominator by a factor equal to 1.960/1.645. We chose a 95 % confidence as opposed to the 90 % confidence that is released by the ACS in order to reduce the likelihood of false inference from the number of comparisons of each indicator using the three survey cycles. All calculations were derived using ACS guidelines for constructing proportions and ratios (US Census Bureau 2009). The calculation steps for each indicator are listed in Table 1. The indicators we evaluated were selected based on their previous application for monitoring drowning-related injuries in the US (Brenner et al. 2001; Browne et al. 2003; Gilchrist & Parker 2014; Peek-Asa et al. 2004; Saluja et al. 2006; Thompson & Rivara 2000; Wright et al. 2013) and abroad (Burrows et al. 2013; Giashuddin et al. 2009; Kim et al. 2007) All geographic and socioeconomic files were downloaded from the American Fact Finder database.

**Table 1:** Socioeconomic construct description built from the American Community Survey annual and multi-year estimates. File names for calculation based on 2013 ACS data file headings

Construct	Definition	ACS data file	Calculation
% US residency less than 1 years	Percent of population living abroad one year ago	DP02	Abroad [HC01_VC126] / Population 1 year and over [HC01_VC119]
% College/postgraduate degree	Percent of persons, age 25 years and older, with an associate’s, bachelor’s, graduate or professional degree	DP02	(Associate’s degree [HC01_VC90] + bachelor’s degree [HC01_VC91] + graduate or professional degree [HC01_VC92])/Population 25 years and over [HC01_VC85]
% High school graduates	Percent of population, age 25 years and older, with a high school diploma	DP02	High school graduate (included equivalency) [HC01_VC88]/Population 25 years and over [HC01_VC85]
% Black	Percent of black or African American population	DP05	Black of African American [HC01_VC79] / Total population – race alone or in combination with any other [HC01_VC77]
% Hispanic	Percent of Hispanic or Latino population	DP05	Hispanic or Latino (of any race) [HC01_VC88]/Total population [HC01_VC87]
% Non-white	Percent of non-white population	DP05	(Total population – race alone or in combination with any other [HC01_VC77]– White [HC01_VC78]) / Total population – race alone or in combination with any other [HC01_VC77]
Gini coefficient	A measure of income inequality, reflects the distribution of income (2011 ACS calculations)	B19083	Gini index [HD01_VD01]
Median household income	Median household income (dollars)	DP03	Median household income [HC01_VC85]
% Unemployment	Percent of population, 16 years and older, in the labor force and unemployed	DP03	In the labor force - Unemployed [HC01_VC07]/Employment status [HC01_VC04]
County population	Total population of the county	DP05	(Male [HC01_VC04] + Female [HC01_VC05])

Statistically valid measures of change between consecutive multi-year surveys can be calculated to determine whether ACS estimates for the same geographic areas are statistically significantly different.

The steps required to approximate the difference in standard errors between overlapping surveys are published by the ACS (US Census Bureau 2009) In this study, we report the number and percentage of census areas within each multi-year data cycle having estimates that likely reflect true changes in socioeconomic conditions. Here, we report two different models for evaluating the reliability of overlapping surveys. In the first model, differences in the estimates for the same geographic area were compared using the maximum amount of overlap between adjacent surveys. For example, the maximum amount of overlap for consecutive 5-year survey occurs when using the 2012 and 2013 survey cycles to represent annual change in socioeconomic conditions. This is the only method that allows for annual surveillance of socioeconomic conditions for all US counties. In the second model, we evaluated differences between the estimates using the minimum amount of overlap between consecutive surveys. The minimal amount of overlap occurs when comparing the 2009 and 2013 5-year data cycles, effectively placing a 4-year gap in annual surveillance.

We used negative binomial regression to compare relative risk estimates for each estimate using the annual and multi-year data cycles. All rates were age-adjusted and weighted using the 2000 US standard million population. For each data cycle, relative risk estimates were derived from a single calendar year of drowning-related injury deaths. For example, only drowning-related deaths recorded in 2009 were used when comparing estimates generated from the 2009 annual cycle and the 2009 3-year and 5-year data cycles. Our rationale for comparing estimates for a single calendar year was two-fold. First, our objective was to determine which data cycle and socioeconomic indicator produced the greatest number of statistically significantly relative risk estimates over time. Second, if relative risk estimates are similar across survey cycles it would support the use of using a limited but annually updated data file for approximating injury risk on a national scale. The data analysis for this study was generated using SAS software, Version 9.4 of the SAS System for Windows.

## Results

Between 2006 and 2013 there were 28,367 deaths attributed to unintentional drowning and submersions recorded by the NCHS. Thirty-six records were missing the person’s age and were excluded. Nationally, the proportion of drowning deaths during this period was highest among adults aged 45 to 64 (25.3 %) and 25 to 44 (23.5 %) and lowest among children and adolescents aged 5 to 14 (6.7 %) (Table 2). The majority of drowning victims were male (77.5 %) and White non-Hispanic (68.5 %). The numbers of drowning-related deaths are relatively low compared to all-cause injury mortality, accounting for less than 2 % of all-cause injury-related deaths (ICD10: V01–Y36, Y85–Y87, Y89, U01.0-U01.3, U01.5-U01.9, U02, U03) between 2006 and 2013.

**Table 2:** Characteristics of US unintentional drowning and submersion deaths: 2006–2013

	Number (%)
Gender	
Males	21,975 (77.5)
Females	6392 (22.5)
Age, y	
Under 5	3842 (13.6)
5 to 14	1895 (6.7)
15–24	4603 (16.2)
25–44	6658 (23.5)
45–64	7173 (25.3)
Over 64	4160 (14.7)
Race/ethnicity	
White, non-Hispanic	18,377 (64.8)
Black, non-Hispanic	4305. (15.2)
Hispanic	3845 (13.5)
Native American	474 (1.7)
Asian/Pacific Islander	1242 (4.4)
Not specified	124 (0.4)

Source: National Center for Health Statistics Compressed Mortality File, 2006–2013

The proportion of high, moderate, and low CV scores for each ACS estimate for the annual and multi-year data cycles are shown in Table 3. The first column in the table represents the number of census counties for each cycle that had reportable data to build the estimate. The subsequent columns list the percentages of high, moderate, and low reliability CV estimates for each indicator. The first 3-year data cycle was released in 2007 and is derived from 2005–2007 household responses. The first 5-year data cycle was released in 2009 and is derived from 2005–2009 household responses.

**Table 3:** Percentage of census areas (counties) having ‘High Reliability’ (M, CV 12–40 %) and ‘Low Reliability’ (L, >40 %) Coefficient of Variation (CV), annual, 3 years and 5-years American Community Survey (ACS) data cycles (95 % confidence level)

	2006				2007				2008				2009				2010				2011				2012				2013			
	n	H	M	L	n	H	M	L	n	H	M	L	n	H	M	L	n	H	M	L	n	H	M	L	n	H	M	L	n	H	M	L
Annual ACS data cycle																																
% US residency less than 1 year	746	1.7	35.9	62.3	745	1.6	36.0	62.4	449	1.7	33.8	64.5	751	1.5	29.7	68.8	767	1.3	33.0	65.7	775	1.3	33.4	65.3	786	2.4	36.3	61.3	791	1.8	36.5	61.7
% college/postgraduate degree	780	99.0	1.0	0.0	787	98.7	1.3	0.0	788	98.9	1.1	0.0	788	99.1	0.9	0.0	803	98.6	1.4	0.0	809	98.6	1.4	0.0	811	99.6	0.4	0.0	812	99.6	0.4	0.0
% high school graduates	780	100.0	0.0	0.0	787	99.6	0.4	0.0	788	99.5	0.5	0.0	788	99.9	0.1	0.0	807	99.8	0.3	0.0	809	99.5	0.5	0.0	811	99.8	0.3	0.0	812	99.5	0.5	0.0
% African American	--	--	--	--	--	--	--	--	--	--	--	--	--	--	--	--	--	*	*	*	--	--	--	--	--	--	--	--	--	--	--	--
% Hispanic	--	--	--	--	--	--	--	--	--	--	--	--	--	--	--	--	--	*	*	*	--	--	--	--	--	--	--	--	--	--	--	--
% non-white	--	--	--	--	--	--	--	--	--	--	--	--	--	--	--	--	--	*	*	*	--	--	--	--	--	--	--	--	--	--	--	--
Gini coefficient	783	99.7	0.3	0.0	788	99.6	0.4	0.0	790	99.8	0.3	0.0	792	99.8	0.3	0.0	807	99.9	0.1	0.0	811	99.5	0.5	0.0	814	99.6	0.4	0.0	817	99.9	0.1	0.0
Median household income	783	99.9	0.1	0.0	788	99.9	0.1	0.0	790	99.9	0.9	0.0	792	99.2	0.8	0.0	807	99.5	0.5	0.0	811	99.1	0.9	0.0	814	99.3	0.7	0.0	817	99.3	0.7	0.0
% Unemployment	776	30.2	69.9	0.0	780	29.1	70.9	0.0	780	28.0	71.8	0.3	792	41.5	58.5	0.0	807	45.2	54.8	0.0	807	39.7	60.4	0.0	813	42.8	57.2	0.0	813	35.9	63.7	0.4
Country population	783	100.0	0.0	0.0	780	100.0	0.0	0.0	789	100.0	0.0	0.0	790	100.0	0.0	0.0	807	100.0	0.0	0.0	810	100.0	0.0	0.0	813	100.0	0.0	0.0	816	100.0	0.0	0.0
3-year ACS data cylce																																
% US residency less than 1 year	*	*	*	*	1698	3.1	31.4	65.6	1711	1.1	18.6	80.3	1702	3.4	31.0	65.6	1696	3.5	30.6	65.9	1721	3.5	30.7	65.8	1728	3.8	32.2	63.9	1745	3.8	32.8	63.4
% college/postgraduate degree	*	*	*	*	1799	95.1	5.0	0.0	1803	95.3	4.7	0.0	1808	96.0	4.0	0.0	1831	96.8	3.2	0.0	1828	97.3	2.7	0.0	1826	98.1	1.9	0.0	1824	99.7	0.3	0.0
% high school graduates	*	*	*	*	1799	99.8	0.2	0.0	1803	99.7	0.3	0.0	1808	99.7	0.3	0.0	1831	99.8	0.2	0.0	1828	99.9	0.1	0.0	1826	99.8	0.2	0.0	1824	99.7	0.3	0.0
% African American	--	--	--	--	--	--	--	--	--	--	--	--	--	--	--	--	--	--	--	--	--	--	--	--	--	--	--	--	--	--	--	--
% Hispanic	--	--	--	--	--	--	--	--	--	--	--	--	--	--	--	--	--	--	--	--	--	--	--	--	--	--	--	--	--	--	--	--
% non-white	--	--	--	--	--	--	--	--	--	--	--	--	--	--	--	--	--	--	--	--	--	--	--	--	--	--	--	--	--	--	--	--
Gini coefficient	*	*	*	*	1817	99.9	0.1	0.0	1822	99.8	0.2	0.0	1823	99.8	0.2	0.0	1843	99.8	0.2	0.0	1844	99.8	0.2	0.0	1846	99.8	0.2	0.0	1847	100.0	0.0	0.0
Median household income	*	*	*	*	1817	99.2	0.8	0.0	1822	99.3	0.7	0.0	1823	99.3	0.7	0.0	1843	99.2	0.8	0.0	1844	99.3	0.7	0.0	1846	99.4	0.6	0.0	1847	99.3	0.7	0.0
% Unemployment	*	*	*	*	1788	35.1	63.7	1.2	1806	40.5	58.4	1.2	1841	52.8	47.1	0.1	1832	46.7	52.4	1.0	1841	52.8	47.1	0.1	1844	54.2	45.7	0.2	1842	52.5	47.4	0.1
Country population	*	*	*	*	1804	100.0	0.0	0.0	1809	100.0	0.0	0.0	1811	100.0	0.0	0.0	1832	100.0	0.0	0.0	1835	100.0	0.0	0.0	1834	100.0	0.0	0.0	1833	100.0	0.0	0.0
5-year ACS data cycle																																
% US residency less than 1 year	*	*	*	*	*	*	*	*	*	*	*	*	2691	4.9	30.4	64.7	2686	4.1	27.3	68.6	2745	4.4	26.8	68.7	2744	4.2	28.5	67.3	2748	5.2	31.3	63.5
% college/postgraduate degree	*	*	*	*	*	*	*	*	*	*	*	*	3103	88.9	13.1	0.0	3100	87.1	12.9	0.1	3108	88.3	11.7	0.1	3109	90.0	10.0	0.0	3103	91.4	8.5	0.1
% high school graduates	*	*	*	*	*	*	*	*	*	*	*	*	3104	96.8	3.0	0.1	3099	96.8	3.1	0.1	3109	96.9	3.0	0.1	3110	97.0	2.9	0.0	3105	97.2	2.8	0.0
% African American	--	--	--	--	--	--	--	--	--	--	--	--	--	--	--	--	--	--	--	--	--	--	--	--	--	--	--	--	--	--	--	--
% Hispanic	--	--	--	--	--	--	--	--	--	--	--	--	--	--	--	--	--	--	--	--	--	--	--	--	--	--	--	--	--	--	--	--
% non-white	--	--	--	--	--	--	--	--	--	--	--	--	--	--	--	--	--	--	--	--	--	--	--	--	--	--	--	--	--	--	--	--
Gini coefficient	*	*	*	*	*	*	*	*	*	*	*	*	1823	100	0	0	3143	98.8	1.2	0.0	3143	98.8	1.1	0.0	3143	98.8	1.2	0.0	3143	98.8	1.2	0.0
Median household income	*	*	*	*	*	*	*	*	*	*	*	*	3143	95.4	4.6	0.1	3143	95.3	4.7	0.0	3143	95.2	4.7	0.1	3143	95.3	4.7	0.0	3143	95.9	4.1	0.0
% Unemployment	*	*	*	*	*	*	*	*	*	*	*	*	3132	35.6	56.3	8.1	3131	39.3	54.1	6.6	3133	42.2	51.8	6.0	3136	44.5	50.0	5.4	3139	47.0	48.2	4.8
Country population	*	*	*	*	*	*	*	*	*	*	*	*	3096	99.1	0.9	0.0	3097	99.0	0.9	0.0	3111	99.1	0.8	0.0	3121	99.2	0.8	0.0	3116	99.4	0.6	0.0

--estimate not compared because the estimate is controlled

*ACS data cycle not available

Five of the ten indicators within the annual ACS data cycle, including *% college/postgraduate degree, % high school graduates, Gini coefficient, median household income,* and *county population,* generated high reliability CV estimates for at least 98 % of all counties each year between 2006 and 2013. These same indicators generated high reliability estimates for at least 95 % of all census counties for the 2007–2013 3-year file. Within the 5-year cycle, the proportion of census areas with high reliability CV estimates was reduced, but none of the same indicators generated high reliability CV estimates for less than 85 % of all counties. Across all cycles, the proportion of ‘low reliability’ CV estimates was greatest for the indicator *% US residency less than 1 year*, with at least 60 % of all census areas for all data cycles having low reliability. The CV for the remaining indicators could not be compared because all are released as controlled estimates.

Table 4 shows the percentage of the same geographic areas having estimates that can be considered statistically different (*p* < 0.05) between sequential and semi-sequential multi-year data cycles. Of all indicators and across all configurations, changes in county population were the least likely to be attributed to sampling error. Among the other indicators, the likelihood that the observed differences in the estimate was attributed to real changes in area socioeconomic conditions varied. For example, changes in area unemployment rates between consecutive 3-year surveys were statistically significantly different in as few as 13 % of all counties to as many as 89 % of all counties depending on survey year.

**Table 4:** number and percentage of all census areas (US counties) having statistically significantly different estimates for the same geographic areas across consecutive and non-consecutive multi-year data cycles (95 % confidence level)

	ACS 3-year data cycles														ACS 5-year data cycles									
	Maximum overlap								Minimum overlap						Maximum overlap								Miximum overlap	
	2009 vs 2010		2010 vs 2011		2011 vs 2012		2012 vs 2013		2009 vs 2011		2010 vs 2012		2011 vs 2013		2009 vs 2010		2010 vs 2011		2011 vs 2012		2012 vs 2013		2009 vs 2013	
	N	%	N	%	N	%	N	%	N	%	N	%	N	%	N	%	N	%	N	%	N	%	N	%
US residency less than 5 years	132	7.1	146	7.9	131	7.1	130	7.0	113	6.1	100	5.4	69	3.7	267	8.5	340	10.8	268	8.5	321	10.2	165	5.2
College/postgraduate degree	167	9.0	126	6.8	136	7.4	168	9.1	162	8.7	171	9.2	206	11.0	464	14.8	311	9.9	320	10.2	367	11.7	713	22.7
High school graduates	258	14.0	136	7.4	117	6.3	152	8.2	197	10.7	125	6.8	244	13.2	430	13.7	434	13.8	376	12.0	275	8.7	570	18.1
African American	--	--	--	--	--	--	--	--	--	--	--	--	--	--	--	--	--	--	--	--	--	--	--	--
Hispanic	--	--	--	--	--	--	--	--	--	--	--	--	--	--	--	--	--	--	--	--	--	--	--	--
Non-white	--	--	--	--	--	--	--	--	--	--	--	--	--	--	--	--	--	--	--	--	--	--	--	--
Gini coefficient	93	5.0	98	5.3	115	6.2	131	7.1	339	18.4	376	376	20.3	150	8.1	166	5.3	217	6.9	247	321	10.2	166	5.3
Median household income	168	9.1	139	7.5	130	7.0	155	8.4	489	26.5	1144	61.9	175	9.5	543	17.3	705	22.4	356	11.3	266	8.5	797	25.4
Unemployment	766	41.4	656	35.5	243	13.1	1642	88.8	973	52.7	497	26.9	1303	70.5	975	31.0	875	27.8	658	20.9	2928	93.2	2601	82.8
County population	1351	73.1	681	36.8	685	37.0	664	35.9	1281	69.4	813	44.0	805	43.5	2366	75.3	1253	39.9	1233	39.2	1205	38.3	2104	66.9

For both multi-year data cycles, using the minimum number of years of overlap between survey years improved the reliability of the estimates. For the 3-year data cycle, changes in socioeconomic characteristics for approximately 25 % of all census counties (range 4 to 70 %) were attributed to real changes in area conditions when survey cycles were used every 2 years, compared to an average of 17 % of all counties (range 5 to 89 %) when survey cycles were used consecutively. Similarly, changes in socioeconomic characteristics for approximately 32 % (range 5 to 83 %) of all census areas in the 5-year file were attributed to real changes in area conditions when there was a 4-year lag between survey years compared to an average of 21 % of all counties (range 5 to 93 %) when survey cycles were used consecutively.

Table 5 shows age-adjusted relative risk estimates in drowning-related deaths across the US from 2006–2013 using all ten indicators. The indicators are partitioned by survey year as well as data cycle. Due to the release of the first 5-year data cycle in 2009 there is 1 year of overlap in the comparison of the 5-year data cycles. Annual mortality estimates for 2005 were excluded because the same person file (DP05) used to construct the age-adjusted rates was not publically available until 2006. Deaths occurring in census counties having no recorded population denominator were excluded.

**Table 5:** Comparison of relative risk (RR) estimates for related injury mortality rates across ACS survey cycles, 2006–2013

Socioeconomic indicator	ACS data Cycle^a^	2006	2007	2008	2009	2010	2011	2012	2013
% US residency less than 1 year	Annual cycle	1.29 (0.93–1.77)	1.27 (1.01–1.58)	1.42 (1.18–1.171)	1.05 (0.66–1.66)	1.26 (1.07–1.49)	1.22 (0.93–1.59)	1.08 (0.91–1.28)	1.29 (1.15–1.46)
	3-year cycle	--	1.07 (0.92–1.25)	1.15 (1.04–1.28)	1.07 (0.95–1.20)	1.66 (1.14–2.41)	0.98 (0.78–1.21)	0.90 (0.69–1.17)	1.33 (1.16–1.53)
	5-year cycle	--	--	--	1.09 (0.71–1.67)	1.29 (1.14–1.47)	1.05 (0.51–2.19)	0.84 (0.57–1.24)	1.04 (0.71–1.52)
	Annual cycle	1.28 (1.02–1.61)	0.87 (0.76–0.98)	1.05 (0.88–1.25)	1.05 (0.94–1.18)	1.14 (1.01–1.29)	1.01 (0.85–1.19)	1.17 (0.95–1.46)	0.82 (0.73–0.92)
% college/postgraduate degree	3-year cycle	--	0.89 (0.78–1.01)	1.23 (1.10–1.38)	0.92 (0.82–1.02)	0.91 (0.81–1.02)	1.18 (1.05–1.32)	1.13 (0.82–1.57)	0.79 (0.58–1.08)
	5-year cycle	--	--	--	1.16 (1.02–1.31)	1.01 (0.90–1.15)	1.13 (1.01–1.29)	1.31 (0.91–1.87)	0.84 (0.57–1.25)
	Annual cycle	1.02 (0.75–1.40)	0.90 (0.61–1.34)	1.13 (0.64–1.97)	1.55 (1.37–1.74)	1.16 (1.03–1.31)	1.02 (0.90–1.15)	0.88 (0.49–1.56)	1.25 (0.75–2.08)
% high school graduates	3-year cycle	--	1.24 (0.88–1.76)	1.16 (1.04–1.32)	1.30 (0.90–1.88)	1.40 (1.05–1.86)	0.81 (0.67–0.99)	0.86 (0.76–0.97)	1.40 (1.23–1.59)
	5-year	--	--	--	1.31 (1.14–1.49)	1.25 (0.95–1.66)	0.96 (0.75–1.24)	0.91 (0.52–1.60)	1.27 (1.11–1.46)
	Annual cycle	1.08 (0.90–1.30)	1.07 (0.94–1.21)	0.94 (0.65–1.36)	0.88 (0.70–1.09)	1.02 (0.79–1.32)	0.88 (0.74–1.03)	0.86 (0.76–0.98)	0.56 (0.42–0.75)
% African American	3-year cycle	--	1.04 (0.90–1.20)	1.10 (0.78–1.56)	1.25 (0.97–1.61)	1.00 (0.75–1.31)	0.92 (0.81–1.04)	0.94 (0.77–1.15)	0.67 (0.53–0.85)
	5-year	--	--	--	1.08 (0.92–1.27)	0.98 (0.74–1.26)	0.87 (0.78–0.97)	0.71 (0.63–0.79)	0.80 (0.68–0.94)
	Annual cycle	1.56 (1.29–1.89)	1.14 (0.86–1.50)	1.53 (1.04–2.25)	1.58 (1.39–1.79)	1.44 (0.87–2.38)	1.49 (0.77–2.89)	1.09 (0.75–1.57)	1.62 (1.37–1.92)
% Hispanic	3-year cycle	--	1.05 (0.91–1.20)	1.10 (0.97–1.25)	1.39 (1.24–1.56)	1.44 (1.28–1.62)	0.98 (0.87–1.09)	1.06 (0.87–1.30)	1.38 (0.90–2.11)
	5-year cycle	--	--	--	1.27 (1.13–1.43)	1.35 (1.20–1.52)	1.18 (1.05–1.33)	1.13 (0.75–1.69)	1.22 (0.72–2.08)
	Annual cycle	1.26 (1.11–1.43)	1.00 (0.59–1.70)	0.95 (0.84–1.07)	1.08 (0.96–1.22)	1.42 (1.07–1.88)	0.92 (0.62–1.37)	0.79 (0.49–1.26)	1.02 (0.91–1.16)
% non-white	3-year cycle	--	1.30 (0.78–2.18)	1.15 (0.84–1.57)	1.47 (0.76–2.85)	1.34 (0.91–1.96)	0.83 (0.74–0.94)	0.82 (0.68–1.00)	1.09 (0.77–1.53)
	5-year cycle	--	--	--	1.24 (1.11–1.39)	1.44 (0.88–2.32)	0.86 (0.77–0.97)	1.02 (0.91–1.15)	1.04 (0.61–1.79)
	Annual cycle	0.74 (0.52–1.07)	1.13 (1.00–1.28)	0.95 (0.54–1.67)	1.09 (0.79–1.50)	1.31 (1.04–1.65)	0.93 (0.71–1.22)	1.37 (1.00–1.86)	1.24 (0.80–1.92)
Gini coefficient	3-year cycle	--	1.25 (1.11–1.41)	1.25 (0.97–1.61)	1.36 (0.77–2.40)	1.38 (0.77–2.48)	1.02 (0.55–1.93)	1.18 (1.06–1.33)	1.19 (0.92–1.54)
	5-year	--	--	--	1.45 (0.80–2.62)	1.35 (0.80–2.26)	1.10 (0.71–1.70)	1.09 (0.98–1.22)	1.24 (1.11–1.38)
	Annual cycle	0.84 (0.56–1.28)	0.63 (0.40–0.98)	0.98 (0.81–1.20)	1.06 (0.88–1.30)	1.09 (0.94–1.27)	1.17 (1.04–1.32)	1.29 (0.95–1.74)	0.73 (0.49–1.08)
Median household income	3-year cycle	--	0.78 (0.56–1.08)	1.22 (0.84–1.77)	1.21 (0.71–2.05)	1.05 (0.86–1.27)	1.30 (1.09–1.54)	1.23 (1.09–1.54)	0.67 (0.42–1.09)
	5-year cycle	--	--	--	1.17 (0.95–1.45)	1.00 (0.83–1.20)	1.21 (1.09–1.37)	1.24 (0.92–1.67)	0.74 (0.60–0.92)
	Annual cycle	0.83 (0.39–1.78)	0.62 (0.31–1.26)	1.73 (0.94–3.16)	0.99 (0.53–1.85)	1.77 (0.88–3.57)	1.70 (0.91–3.16)	1.24 (0.65–2.36)	0.87 (0.47–1.62)
% Unemployment	3-year cycle	--	0.75 (0.55–1.03)	1.24 (1.12–1.38)	1.09 (0.82–1.44)	1.21 (1.01–1.44)	0.97 (0.67–1.40)	1.19 (1.02–1.39)	0.93 (0.83–1.04)
	5-year cycle	--	--	--	0.74 (0.53–1.02)	1.06 (0.92–1.22)	1.12 (0.77–1.61)	1.29 (1.16–1.45)	1.02 (0.87–1.20)
	Annual cycle	0.99 (0.75–1.31)	0.89 (0.44–1.79)	0.94 (0.70–1.26)	1.09 (0.95–1.25)	0.99 (0.86–1.14)	0.89 (0.77–1.03)	1.09 (0.94–1.26)	0.76 (0.46–1.24)
County population	3-year cycle	--	1.07 (0.92–1.25)	0.96 (0.83–1.11)	1.13 (0.67–1.92)	1.10 (0.95–1.27)	1.66 (1.03–2.70)	1.08 (0.55–2.10)	0.72 (0.62–0.85)
	5-year	--	--	--	1.27 (0.63–2.54)	1.35 (0.96–1.90)	1.48 (0.88–2.47)	1.06 (0.72–1.56)	0.86 (0.73–1.03)

^a^All drowning-related mortality rates are based on the calendar year that corresponds with the release of the ACS data cycle

-- ACS data cycle not available

Three results from the relative risk evaluations stand out. First, although some indicators were more predictive of injury disparities than others, including % *Hispanic, % population without a high school diploma,* and % *residency less than one year*, no indicator produced statistically significant estimates consistently over time or across data cycles. Second, the association between the indicators and drowning-related mortality rates did not consistently improve when the number of census areas included in the comparison increased. For example, on average the 3-year data cycle produced the greatest proportion of statistically significant estimates for all indicators and the annual file produced more statistically significant estimates than the 5-year file. Third, the proportion of African American persons per area consistently showed a protective effect of drowning-related mortality rates. This association should be interpreted with caution given the different demographic structure of rural census areas.

## Discussion

We evaluated the reliability of 10 socioeconomic indicators derived from the annual and multi-year ACS data cycles that have previously been employed for monitoring disparities in injury risk. In addition, we assessed the predictability of each indicator for facilitating the detection of differences in injury risk on an annual and semi-annual basis at a national scale using county-level injury mortality data. To our knowledge, this is the first study to evaluate the reliability and predictability of the ACS for drowning-related injury mortality surveillance.

Three key findings from this study stand out. First, only five of the ten indicators produced reliable estimates of area socioeconomic conditions, but these five indicators were consistently reliable across all three data cycles. Second, when overlapping multi-year surveys were used to represent changes in annual socioeconomic conditions fewer than 25 % of all US counties generated estimates that were unlikely to have occurred by chance due to sampling error. Third, no socioeconomic indicator was consistently predictive of disparities in drowning-related mortality over time or across data cycles at the national scale. Taken together, the evidence uncovered by our analysis indicates that improvement in the reliability of the ACS can be made and that continuously predicting disparities in drowning-related injury risk at the national scale using census-level geographic data is not possible using these ten indicators.

Although these findings can be considered somewhat discouraging, in our view the ACS data cycles nonetheless provide two opportunities to advance injury surveillance and data collection. First, the study identified five socioeconomic indicators that are consistently reliable across ACS data cycles: *% college/graduate degree, % high school graduation, Gini coefficient, median household income,* and *county population*. The majority of these indicators are known to be predictive of other injury-related outcomes (Bell et al. 2014). For these indicators, researchers could employ the annual ACS data cycle as a barometer for current socioeconomic conditions, while using the multi-year files to confirm and add precedence to the changing landscape of disparities in injury risk. Second, the indicator *county population* was the most consistently reliable indicator across ACS survey cycles and also the least likely to be effected by sampling error in the overlap analysis. This supports the use of using either an annual or multi-year population file to estimate yearly or semi-yearly changes in drowning-related deaths, which is one of three drowning-related indicators prioritized by the National Center for Injury Prevention and Control (NCIPC) (Thomas & Johnson 2014). By extension, the same indicator could be used to inform the *Healthy People 2020* target of a 10 % reduction target for drowning related deaths over the next decade (U.S. Department of Health and Human Services. Healthy People 2020) However, additional tests are required to determine if the annual file can substitute for either of the multi-year data cycles with respect to monitoring age-adjusted trends relevant to hospitalizations or emergency department visits, both of which are monitored by the NCIPC.

That only a small portion of all census areas generated statistically significant differences between overlapping multi-year surveys suggests that researchers should be judicious if using the yearly released multi-year files to represent annual socioeconomic conditions. While socioeconomic conditions do change over time, that the majority of socioeconomic indicators were not statistically significantly different between overlapping survey years suggests that any observed differences obtained from the ACS could be attributed to sampling error rather than any ‘real’ changes in area unemployment rates, poverty levels, and other demographic changes. This suggests that the best way to employ the multi-year files for injury surveillance is on a semi-annually basis, using the full 3- and 5-year gaps between release years. The results of the regression model lend credence to this finding as the association between the indicators and drowning-related deaths did not increase as a result of increasing the number of census areas in the analysis. Notwithstanding this limitation in the data, access to current socioeconomic information – even if limited to the annual ACS data file – is a significant strength of the survey as it brings greater currency to efforts to monitor trends, (Injury Surveillance Workgroup. Consensus Recommendations for Using Hospital Discharge Data for Injury Surveillance. State and Territorial Injury Prevention Directors Association & Marietta (GA) 2003; Kozak et al. 2004) improves upon the information required for regional policy and planning for about prevention needs, (Centers for Disease Control 1998; Centers for Disease Control 1999; Hiller et al. 2009; Mireles et al. 2015) and improves access to information that can impact legislation to improve civic safety (Everett et al. 1997; Helmkamp et al. 2012; Pressley et al. 2009).

Importantly, findings from this study must be viewed within the context of three key limitations. Firstly, the evaluation of injury risk was limited to mortality records. While evidence does show that indicators of injury mortality are similarly indicative of injury morbidity (Bell et al. 2014) additional evaluations of hospitalization data against these ACS indicators are needed. Secondly, we included only area socioeconomic indicators in our evaluation, excluding other important covariates known to lead to disparities in drowning-related injury risk, including alcohol consumption (Petridou & Klimentopoulou 2006) the use of safety device, (Treser et al. 1997) and duration of rescue (Topjian et al. 2012). These limitations stem from the lack of documentation in national mortality records. Although this information is obtainable from databases such as the National Trauma Registry (NTR), the evaluation would have to be completed on a state-by-state basis using hospital discharge abstracts because the NTR does not release fine-scale geographic identifiers at the patient-level. Thirdly, the study was limited to census counties as opposed to smaller geographic scales, such as zip codes, census tracts, or block faces. Previous studies have shown that differences in risk attributable to socioeconomic position, or status, are typically largest when viewed using census tracts or blocks (Hameed et al. 2010; Krieger et al. 2003). However, fine-scale evaluation of the ACS would require state-by-state evaluations as the NCHS does not release US mortality data at a geographic scale below Census County. Within the context of this study’s objectives, US county boundaries are the smallest geographic unit available to assess the reliability and predictability of socioeconomic indicators derived from all three ACS data cycles.

## Conclusion

Despite being highly preventable, injuries are the leading cause of death in the US among all persons under the age of 44 and also the cause of morbidity and mortality with the steepest social gradient (Steenland et al. 2004). For decades, the long-form decennial census served as the primary source of socioeconomic information on the injured patient due to the lack of contextual data contained in hospital registries. Although the full impact of the 2010 replacement of the long-form census with the ACS is still being determined, evidence thus far has shown key weaknesses of the survey. To date, there has been no report on the reliability of the ACS for injury surveillance despite considerable versatility in its application (Aytur et al. 2013; Curry et al. 2015; Hong et al. 2015; Huguet et al. 2014; Landy et al. 2011; Perry et al. 2015; Sastry & Gregory 2013). Our study adds much needed information about the reliability of the ACS and provides a guideline for appropriate monitoring strategies using the annual and multi-year ACS data cycles for drowning-related injury surveillance and data collection.
